# Paradoxical Role of Matrix Metalloproteinases in Liver Injury and Regeneration after Sterile Acute Hepatic Failure

**DOI:** 10.3390/cells7120247

**Published:** 2018-12-06

**Authors:** Débora Moreira Alvarenga, Matheus Silvério Mattos, Mateus Eustáquio Lopes, Sarah Cozzer Marchesi, Alan Moreira Araújo, Brenda Naemi Nakagaki, Mônica Morais Santos, Bruna Araújo David, Viviane Aparecida De Souza, Érika Carvalho, Rafaela Vaz Sousa Pereira, Pedro Elias Marques, Kassiana Mafra, Hortência Maciel de Castro Oliveira, Camila Dutra Moreira de Miranda, Ariane Barros Diniz, Thiago Henrique Caldeira de Oliveira, Mauro Martins Teixeira, Rafael Machado Rezende, Maísa Mota Antunes, Gustavo Batista Menezes

**Affiliations:** 1 Center for Gastrointestinal Biology, Departamento de Morfologia, Instituto de Ciências Biológicas, Universidade Federal de Minas Gerais, Belo Horizonte, Minas Gerais 31270-901, Brazil; deboraalvarengabio@gmail.com (D.M.A.); mattosms@yahoo.com.br (M.S.M.); mateuslopes_12@hotmail.com (M.E.L.); sarahcmarchesi@gmail.com (S.C.M.); amoreiradearaujo@gmail.com (A.M.A.); brendanaemi22@gmail.com (B.N.N.); viviane_lacerda@outlook.com (V.A.D.S.); erikacarvalhobio@gmail.com (É.C.); kassiana93@gmail.com (K.M.); hortsmaciel@gmail.com (H.M.d.C.O.); camiladutra_mm@hotmail.com (C.D.M.d.M.); abarrosdiniz@gmail.com (A.B.D.); maisaantunes@gmail.com (M.M.A.); 2 Departamento de Biologia Animal, Universidade Federal De Viçosa, Viçosa 36570-900, Brazil; monicamoraissantos@gmail.com; 3 Department of Physiology and Pharmacology, University of Calgary, Calgary, AB T2N 4N1, Canada; brunaraujodavid@gmail.com; 4 The Hospital for Sick Children, Toronto, ON M5G 0A4, Canada; rafaelavazsp@gmail.com (R.V.S.P.); pempsufmg@gmail.com (P.E.M.); 5 Departamento de Bioquímica e Imunologia, Laboratório de Imunofarmacologia, Universidade Federal de Minas Gerais, Belo Horizonte, Minas Gerais 31270-901, Brazil; thiagohcoliveira@gmail.com (T.H.C.d.O.); mmtex.ufmg@gmail.com (M.M.T.); 6 Ann Romney Center for Neurologic Diseases, Brigham and Women’s Hospital, Harvard Medical School, Boston, MA 02115, USA; rafaelmachadorezende@gmail.com

**Keywords:** neutrophil, matrix metalloproteinases, liver injury, sterile inflammation

## Abstract

Acetaminophen (APAP) poisoning is one of the leading causes of acute hepatic failure and liver transplantation is often the only lifesaving alternative. During the course of hepatocyte necrosis, an intense accumulation of neutrophils is often observed within the liver microenvironment. Despite the classic idea that neutrophil accumulation in tissues causes collateral tissue damage, there is a growing body of evidence showing that neutrophils can also orchestrate the resolution of inflammation. In this work, drug-induced liver injury was induced by oral administration of APAP and pharmacological intervention was made 12 h after this challenge. Liver injury and repair kinetics were evaluated by a novel combination of enzyme quantifications, ELISA, specific antagonists of neutrophil enzymes and confocal intravital microscopy. We have demonstrated that neutrophil infiltration is not only involved in injury amplification, but also in liver tissue repair after APAP-induced liver injury. In fact, while neutrophil depletion led to reduced hepatic necrosis during APAP poisoning, injury recovery was also delayed in neutropenic mice. The mechanisms underlying the neutrophil reparative role involved rapid degranulation and matrix metalloproteinases (MMPs) activity. Our data highlights the crucial role of neutrophils, in particular for MMPs, in the resolution phase of APAP-induced inflammatory response.

## 1. Introduction

Drug-induced liver injury (DILI) is a serious condition that can cause acute liver failure [1]. Treatment with *N*-acetyl cysteine may be efficient in the first hours, particularly when acetaminophen (APAP) was abused [2], but, usually, liver transplantation is the only lifesaving alternative. APAP poisoning accounts for approximately 50% of acute liver failures in Europe and the United States [3] and, although it is usually safe in therapeutic doses, the elevated number of APAP-induced liver injury cases might be attributable to its high availability and easy access over-the-counter. APAP-induced liver injury may occur in a two hit fashion: (i) After the absorption of excessive amounts, APAP is metabolized in the liver into the toxic intermediate *N*-acetil-p-benzoquinone imine (NAPQI) causing oxidative stress and hepatocyte death by necrosis; (ii) dead hepatocytes release intracellular contents to the extracellular milieu, which act as damage-associated molecular patterns (DAMPs) and trigger robust acute inflammation [4].

Acute inflammation during sterile tissue damage may be crucial to restore tissue homeostasis; however, overt inflammatory reactions are associated with tissue destruction and loss of function. In fact, damaged tissues are rapidly invaded by neutrophils [5], which orchestrate the subsequent steps of acute inflammation, promoting recruitment of additional waves of granulocytes and monocytes [6]. Neutrophils are preloaded with several granules in their cytoplasm, which may rapidly convert these cells from passive circulating leukocytes into potent effectors of acute inflammation. There are four types of cytoplasmic granules in neutrophils, which are named after their schedule of appearance during the maturation period: primary, secondary, tertiary and quaternary granules. Primary granules contain myeloperoxidase (MPO) and are also known as peroxidase-positive granules. The other granules are known as peroxidase-negative granules and contain several proteins, including matrix metalloproteinases (MMPs) [7]. MMPs can exert a plethora of actions, including degradation of the extracellular matrix components, such as collagens, proteoglycans and fibronectin [8,9]. In this context, MMPs play a key role in creating room within interstitial structures that will allow a rapid and precise influx of neutrophils and other immune cells into inflamed tissues.

There is a growing body of evidence demonstrating that neutrophil infiltration within an inflamed liver is associated with additional tissue damage and an enhanced chance of organ failure [5,10,11]. In fact, overt neutrophilic response has a high potential to drive tissue destruction since these cells undergo degranulation to release MMPs, MPO and other enzymes that boost oxidative stress. Therefore, initiation and propagation of acute inflammation are the most investigated effector function of neutrophils. However, how such orchestrated neutrophil infiltration contributes to the resolution of inflammation and the repair of tissue is still largely unknown.

Here we demonstrated, for the first time, that neutrophil infiltration is not only involved in the establishment and amplification of liver injury, but that it can also play a fundamental role in tissue repair after sterile liver injury. Although neutrophil depletion or blockage of neutrophil-mediated damage has been associated with liver injury protection, we showed that neutropenia induction, or the blockage of neutrophil effector functions, 12 h after APAP challenge did not interfere with the liver injury but significantly delayed hepatic tissue repair and recovery of function. Amongst different effector functions that we experimentally inhibited, neutrophil-matrix metalloproteinases emerged as essential to hepatic tissue recovery. Together, our data highlights the crucial role of neutrophil-secreted MMPs in the resolution phase of APAP-induced liver injury.

## 2. Materials and Methods

### 2.1. Animals

Ten- to 12-week-old female C57BL/6J mice were purchased from Centro de Bioterismo in Universidade Federal de Minas Gerais (CEBIO-UFMG, Belo Horizonte, Brazil). Female mice were chosen because, in our experience, male mice had enhanced susceptibility to APAP-mediated injury, precluding our investigation under higher dose protocols. Male mice usually had a similar response to treatments compared to females, but with a higher mortality rate. All animals were housed in conditions controlled for temperature (24 °C) and light and dark cycles of 12:12 h, with food and water ad libitum. The animal Care and Use Committee at UFMG approved all animal studies in this work (CEUA 331/2015).

### 2.2. Acetaminophen-Induced Liver Injury Model

Mice were fasted for 12–15 h before APAP administration (600 mg/kg) or vehicle. Acetaminophen (Sigma-Aldrich, St. Louis, MO, USA) was dissolved in a warm 0.9% saline solution at a concentration of 75 mg/mL, prior gavage. Liver fragments were collected for histology (hematoxylin and eosin staining) and myeloperoxidase activity assay.

### 2.3. Alanine Aminotransferase Assay

Serum alanine aminotransferase (ALT) activity was performed, utilizing a kinetic test (Bioclin), as indicative of liver injury. Briefly, blood samples were harvested and centrifuged at 1.500 g for 10 min. Enzimatic assay were carried out utilizing pure serum samples and three different dilutions (1:10, 1:20, and 1:30). Substrates were added to the serum samples (Hepes buffer pH 7.8, LDH, l-Alanine, and NaCl and sodium azide) and coenzymes (alfacetoglutarate, NADH, and sodium azide). Both were contained in the kit at 37 °C. After 50 s, the plate with the samples was read in a spectrophotometer at 37 °C (340 nm) every 1 min during 4 min.

### 2.4. Indocyanine Green Clearance Rate Assay

To evaluate if liver injury led to hepatic dysfunction, we measured the indocyanine green (ICG) clearance rate from systemic circulation. ICG (Sigma-Aldrich) was administered intravenously (20 mg/kg) and, after 20 min, blood samples were harvested, centrifuged at 1500 g for 10 min, and the serum was used to quantify systemic ICG levels. Samples were seeded in 96 well microplates and quantified in a spectrophotometer (VERSAmax, Molecular Devices, San Jose, CA, USA) at 800 nm.

### 2.5. Histological Analysis

Liver was harvested and fixed in 4% formaldehyde in Phosphate Buffer Saline, pH 7.4 (PBS), dehydrated in alcohol and embedded in paraffin. Then, sections of 4 µm thicknesses were made in microtome, stained with hematoxylin and eosin (HE). Hepatic injury was analyzed utilizing a Nikon Ti microscope through a 40× objective. The area of necrosis was measured as a percentage of the total area analyzed.

### 2.6. Confocal Intravital Imaging

In vivo imaging was performed as previously described [12]. Briefly, mice were anesthetized intraperitoneally (i.p.) with a ketamine and xylazine solution (Syntec, São Paulo, Brazil, 60 mg/kg and 15 mg/kg, respectively) and a midline laparoscopy was performed to expose the liver for imaging. Prior to surgery, mice were injected, intravenously (i.v.), with Phycoerythrin (PE)-conjugated anti-Ly6G (4 µg, eBioscience, San Diego, CA, USA clone 1A8) and Sytox Green (5 nmol/mouse of Invitrogen). Labeled antibodies and florescent probes were diluted in a total volume of 100 µL before injection. Mice were imaged using a Nikon Eclipse Ti with an A1R confocal microscope loaded with a spectral detector and XYZ motorized stage.

### 2.7. Neutrophil Depletion and Pharmacological Interventions

In order to deplete neutrophils, mice received an i.v. injection of Anti-Ly6G (400 µg/mouse, 1A8 clone; eBiosciences) 12 h after the APAP challenge. We also performed different pharmacological intravenous interventions, always 12 h after the APAP challenge. To prevent neutrophil degranulation, mice were treated with: (i) cromoglycate (Sigma-Aldrich, St. Louis, MO, USA) in crescent doses of 5, 50 and 100 mg/kg; (ii) a protease inhibitor cocktail (Sigmafast^TM^, St. Louis, MO, USA, EDTA-Free) in doses of 0.28, 1.4 and 2.8 mg/mouse; (iii) MPO inhibitor 4-aminobenzoic acid hydrazide (4-ABAH, Aldrich Chemistry, St. Louis, MO, USA), 80 mg/kg; (i.v.) matrix metalloproteinases inhibitors (MMP Inhibitor I, Calbiochem), 2 mg/kg; and (v) noncompetitive allosteric CXCR1/2 receptor antagonists (DF2156A, Dompé Farmaceutici SpA, L’Aquila AQ, Italy), 30 mg/kg. All inhibitors were tested alone before challenged with APAP and only doses that caused no injury were used in this study (data not shown).

### 2.8. Neutrophil Myeloperoxidase Activity Assay

MPO assay was performed by adding 25 µL of processed tissue supernatant in 25 μL of 3,3′-5,5′-tetramethylbenzidine (TMB Sigma, St. Louis, MO, USA) diluted in dimethyl sulfoxide (DMSO; Merck, Darmstadt, Germany) at a final concentration of 1.6 mM. After 5 min at 37 °C, 100 µL of PBS supplemented with 6.6 mM of H_2_O_2_ were added followed by a new incubation at 37 °C for 5 min. The reaction was stopped with 100 µL of H_2_SO_4_ 1 M and quantified in a spectrophotometer (VERSAmax, Molecular Devices, San Jose, CA, USA) at 450 nm.

### 2.9. Statistical Analysis

Experimental data analysis was performed with a one-way analysis of variance (Anova with Newman-Keuls post-test) in Prism 6.0 software (GraphPad). All data are given as the mean ± SEM. In vivo experimental groups had six mice per group. Data shown are representative of at least two independent experiments. Differences were considered significant at *p* < 0.05

## 3. Results

### 3.1. Neutrophil Recruitment Kinetics Correlate with Liver Injury

**Figure 1:**
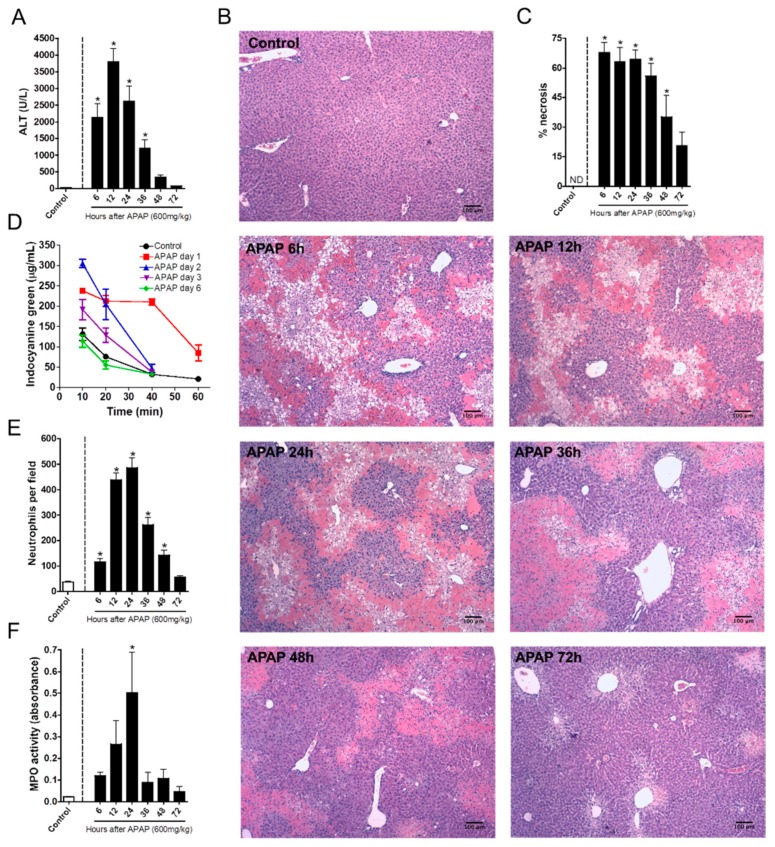
Kinetics of neutrophil influx and acetaminophen (APAP)-induced liver injury. (**A**) Kinetics of serum alanine aminotransferase (ALT) levels. (**B**) Liver histology at different time points after APAP challenge; coloration Hematoxylin & Eosin (HE); images were taken utilizing the 40× objective; scale bar = 100 µm. (**C**) Necrosis score. (**D**) Indocyanine green clearance rate to asses liver function. (**E**) Kinetics of neutrophil influx into the liver after APAP challenge. (**F**) Myeloperoxidase activity assay in liver parenchyma samples evidencing the presence of neutrophils. * *p* ≤ 0.05 (mean ± SEM).

We first investigated the kinetics of liver injury after a single dose of APAP and correlated this with the dynamics of neutrophil migration within the hepatic tissue. APAP overdose (600 mg/kg) caused liver necrosis in a time-dependent manner, which was monitored by increased ALT (alanine aminotransferase) levels in sera. Liver injury started as quickly as 6 h after APAP administration, peaking 12 h later (Figure 1A). Histopathological analyses confirmed the massive liver injury assessed by transaminase activity, also revealing a prominent leukocyte infiltration at these time points. Forty-eight hours after APAP administration, necrotic areas were significantly smaller, suggesting that tissue repair had already started (Figure 1B–C). To further evaluate hepatic function, we measured the liver’s ability to depurate indocyanine green (ICG) in vivo. The ICG clearance rate was lower 24 h after APAP and began to increase at the 48 h mark, reaching a normal depuration rate six days after APAP challenge (Figure 1D). These data suggest that APAP overdose induced not only a massive liver injury, but also hepatic dysfunction that was detectable for at least three days.

To better understand the kinetics of the liver’s inflammatory response during APAP challenge, we investigated the time course of neutrophil migration into the liver by intravital microscopy and confirmed the findings by quantifying MPO activity in the liver samples. Intravital imaging revealed that a minor population of neutrophils naturally resides within hepatic microvasculature under homeostatic conditions. However, after APAP administration, neutrophils rapidly accumulated in the liver parenchyma during the first 12 h after APAP administration; this was sustained until the 24th hour. The number of hepatic neutrophils dramatically decreased at the 36th hour, reaching baseline levels 72 h after challenge (Figure 1E). Importantly, MPO activity followed the kinetics of neutrophil accumulation within the liver (Figure 1F). Taken together, sterile liver injury due to APAP overdose caused hepatic dysfunction in a progressive and time-dependent manner, which matched neutrophil accumulation within the liver microenvironment.

### 3.2. Neutrophil Degranulation Is Crucial to Tissue Repair after APAP-Induced Liver Injury

**Figure 2:**
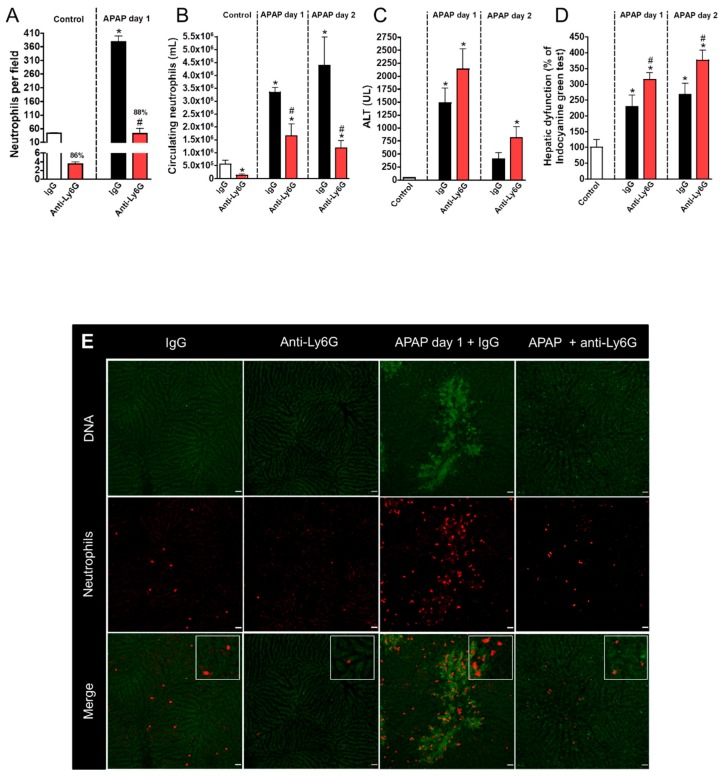
Liver injury evaluation after neutrophil depletion with Anti-Ly6G. (**A**) Neutrophil accumulation in liver parenchyma after APAP (600 mg/kg) and anti-Ly6G (400 µg/mouse) administration. (**B**) Number of circulating neutrophils. (**C**) Serum ALT levels evidencing liver injury. (**D**) Indocyanine green clearance rate. (**E**) Intravital microscopy confirming neutrophil depletion. Red—Phycoerythrin (PE)-conjugated anti-Ly6G (4 µg/mouse); green—Sytox Green (5 nmol/mouse). Scale bar = 50 µm. * *p* ≤ 0.05 vs. control; # *p* ≤ 0.05 vs. IgG with APAP group (mean ± SEM).

Once we established that neutrophils accumulated in the liver after APAP challenge, we hypothesized that these cells participated in the initial steps of tissue recovery. To investigate this, we challenged mice with APAP and depleted neutrophils by using an anti-Ly6G antibody 12 h after APAP challenge, when the injury was already established. This strategy avoided any putative interference in injury course caused by the neutrophil absence. Mice that received the anti-Ly6G antibody had a major decrease in liver neutrophils (about 90% reduction), as assessed by intravital microscopy under baseline conditions and after APAP challenge (Figure 2A,E). Anti-Ly6G treatment also reduced the number of circulating neutrophils 24 and 48 h after APAP challenge (Figure 2B). Strikingly, neutrophil depletion using our protocol had no effect on liver injury on day one after APAP, as highlighted by the similar ALT serum levels in these animals (Figure 2C). However, impaired liver depurative function (Figure 2D) was still observed. Thus, these data suggest that our depletion protocol did not interfere with liver injury progression. However, neutrophil depletion was associated with worse liver function at later time points, suggesting a delay in the recovery phase after APAP injury.

**Figure 3:**
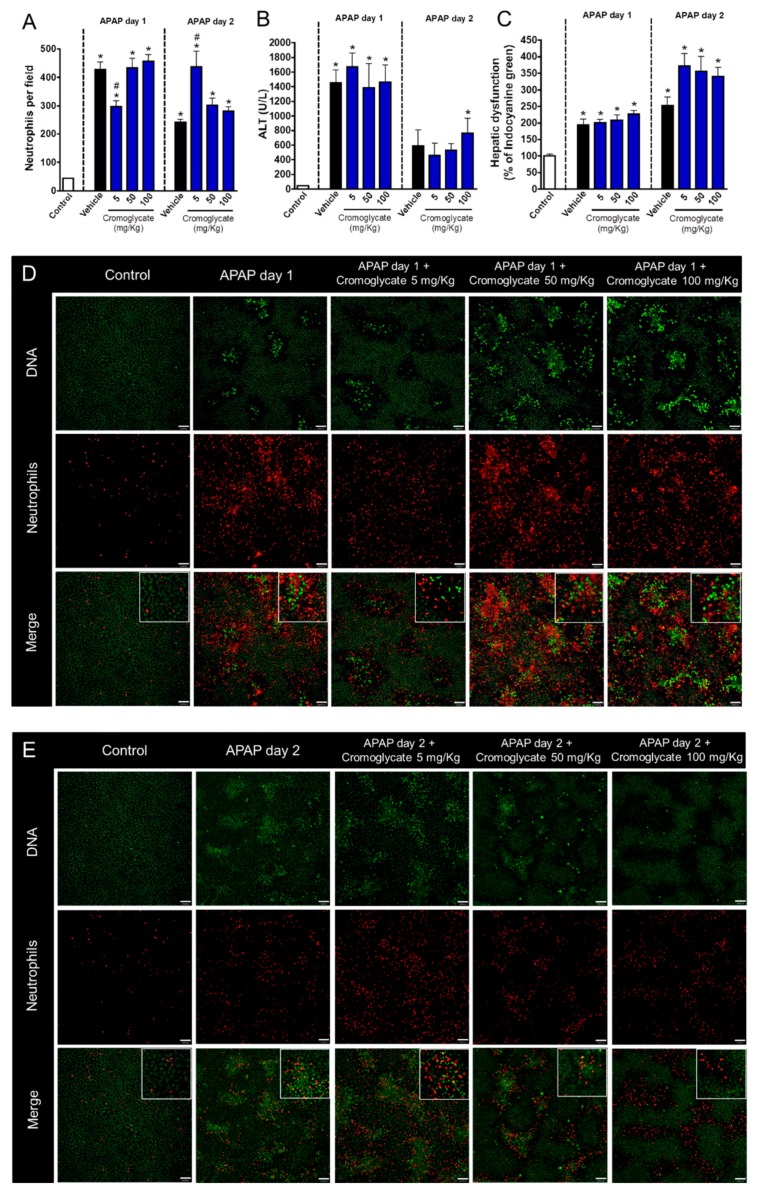
Degranulation blockage delays liver tissue repair. (**A**) Quantification of neutrophils in liver parenchyma. (**B**) Serum ALT levels. (**C**) Hepatic dysfunction assessed by ICG depuration rate. (**D**) Intravital confocal microscopy evidencing neutrophils in liver parenchyma 24 h after APAP administration. (**E**) Intravital confocal microscopy evidencing neutrophils in liver parenchyma 48 h after APAP administration. Green—Sytox Green (5 nmol/mouse); red—phycoerythrin-conjugated anti-Ly6G (4 µg/mouse). Scale bar = 50 µm. * *p* ≤ 0.05 vs. control; # *p* ≤ 0.05 vs. vehicle (mean ± SEM).

We next investigated which neutrophil components were involved in the hepatic regeneration phase after APAP-induced liver injury. It is known that intracellular granules are the main effector mechanism for neutrophils; thus, we first pharmacologically blocked neutrophil degranulation with different doses of cromoglycate (5, 50, and 100 mg/kg), a potent cell membrane stabilizer. Again, drug intervention was performed at the peak of APAP-mediated liver injury (12 h post administration). We found that a lower dose of cromoglycate (5 mg/kg) reduced neutrophil influx 24 h after APAP challenge; however, neutrophils remained adhered to liver parenchyma until later time points (48 h), concomitant with the beginning of the tissue repair phase (Figure 3A,D,E). Although we did not find significant changes in serum ALT levels (Figure 3B), inhibition of neutrophil degranulation by cromoglycate delayed the recovery of liver function, as assessed by the depurative ratio of indocyanine green (Figure 3C). Thus, infiltrated neutrophils may be involved in liver tissue repair and in the restorative mechanisms of liver function.

### 3.3. Neutrophil Proteases, but Not Myeloperoxidase, Participate in Liver Repair after APAP-Induced Injury

**Figure 4:**
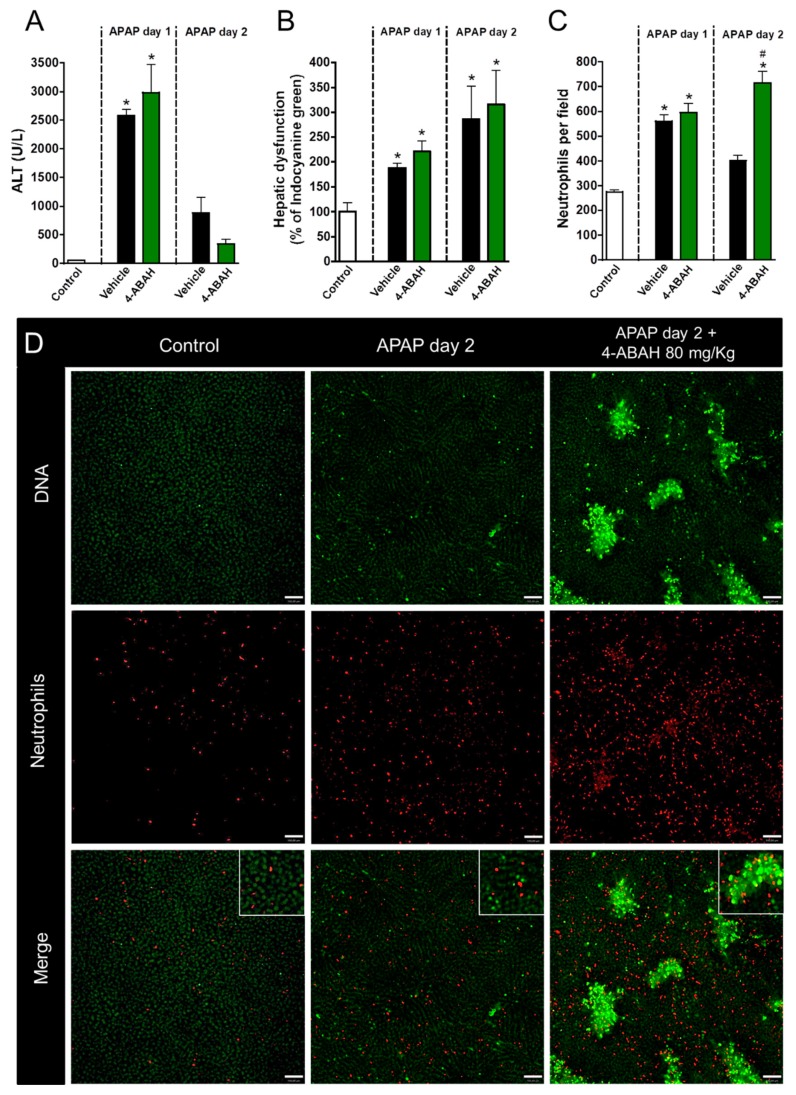
Myeloperoxidase inhibition has no impact on liver repair. (**A**) Serum ALT levels evidencing APAP-induced liver injury. (**B**) Hepatic dysfunction assessed by ICG depuration rate. (**C**) Neutrophil count in liver parenchyma. (**D**) Intravital confocal microscopy showing neutrophil and lesion sites in liver after APAP administration. Green—Sytox Green (5 nmol/mouse); red—phycoerythrin-conjugated anti-Ly6G (4 µg/mouse). Scale bar = 50 µm. * *p* ≤ 0.05 vs. control; # *p* ≤ 0.05 vs. vehicle (mean ± SEM).

Next, we dissected which granule components were involved in the acute inflammatory and regenerative phases of APAP-mediated liver injury. To investigate the role of neutrophil myeloperoxidase, one of the main neutrophil-related enzymes, mice were treated with 4-ABAH, a potent MPO inhibitor (80 mg/kg, i.v., 12 h after APAP challenge). Surprisingly, MPO inhibition, even using higher doses of 4-ABAH, had no detectable effects in liver injury (Figure 4A) or disfunction (Figure 4B). However, similar to our findings with the blockage of degranulation, we observed that neutrophils remained within liver parenchyma at later time points (48 h after APAP administration) when MPO was inhibited, while controls already displayed decreased neutrophilic infiltration at this timepoint (Figure 4C,D). These results suggest that the release of granular enzymes may be one of the mechanisms by which neutrophils are removed from necrotic sites, but do not mediate liver injury or repair.

**Figure 5:**
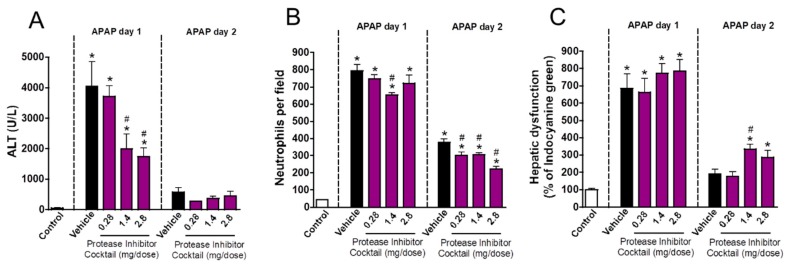
Protease inhibition delayed liver repair. (**A**) ALT levels in serum. (**B**) Number of neutrophils in liver parenchyma. (**C**) Hepatic dysfunction assessed by ICG clearance rate. * *p* ≤ 0.05 vs. control; # *p* ≤ 0.05 vs. vehicle (mean ± SEM).

In addition to potent microbicidal activity, neutrophil proteases have an important role in non-infectious, sterile inflammatory processes. To investigate whether granule proteases are involved in the course of drug-induced liver injury and repair, we treated mice with a protease inhibitor cocktail at crescent doses (0.28, 1.4 or 2.8 mg/mouse). We found that protease inhibition dampened liver injury in a dose-dependent manner (Figure 5A) but had no significant effect on neutrophil accumulation (Figure 5B). In line with our previous findings, proteases inhibition led to an impaired restoration of liver function due to APAP overdose when higher doses were used (1.4 and 2.8 mg/mouse; Figure 5C). Altogether, these data show that amongst granular contents, proteases may have an important role in the pathogenesis of liver injury, but this initial response may be crucial to restore liver function in the post-acute phase of inflammation.

### 3.4. Neutrophil Matrix Metalloproteinases Mediate Liver Function Restoration

**Figure 6:**
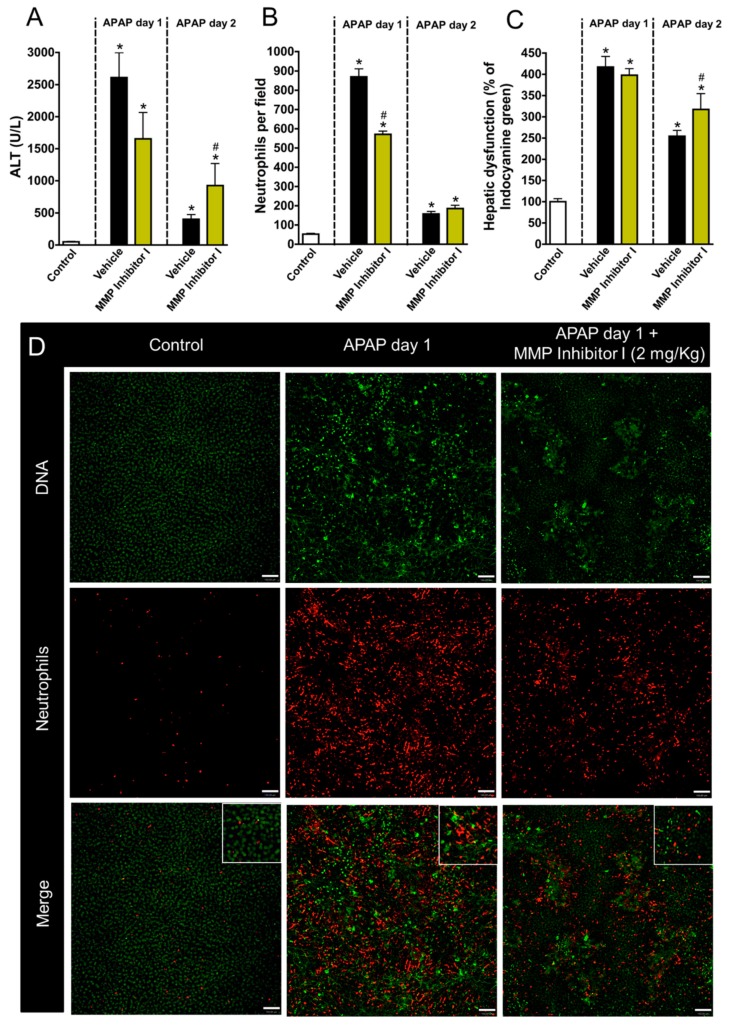
MMPs are important to restore liver function after APAP-induced liver injury. (**A**) ALT levels in serum (**B**) Neutrophils accumulation in liver parenchyma. (**C**) ICG depuration rate. (**D**) Intravital confocal microscopy evidencing liver injury and the presence of neutrophils in liver parenchyma. Green—Sytox Green (5 nmol/mouse); red—phycoerythrin-conjugated anti-Ly6G (4 µg/mouse). Scale bar = 50 µm. * *p* ≤ 0.05 vs. control; # *p* ≤ 0.05 vs. vehicle (mean ± SEM).

To better understand the molecular mechanisms involved in the neutrophil contribution to liver repair, we next blocked different metalloproteinases (MMP1, MMP8, and MMP9) by administering the MMP inhibitor MMP1 (2 mg/kg; i.v.). Of note, this drug is able to exclusively block MMPs in extracellular milieu, not acting on intracellular MMPs, providing an interesting tool to investigate the contribution of secreted MMPs. We found that MMP inhibition dampened liver injury and inflammation after APAP overdose, indicating that secreted MMPs have a detrimental role in sterile liver damage (Figure 6A). This inhibition was also associated with reduced neutrophil migration into the liver (Figure 6B,D). However, MMP inhibition was associated with impaired liver tissue repair and function restoration at later time points (Figure 6C). These data confirmed that, while MMPs can drive liver injury, their release is also critical for liver repair.

### 3.5. Inhibition of Neutrophil Migration Recapitulates the Delay in Liver Repair Observed during MMP Blockage

**Figure 7:**
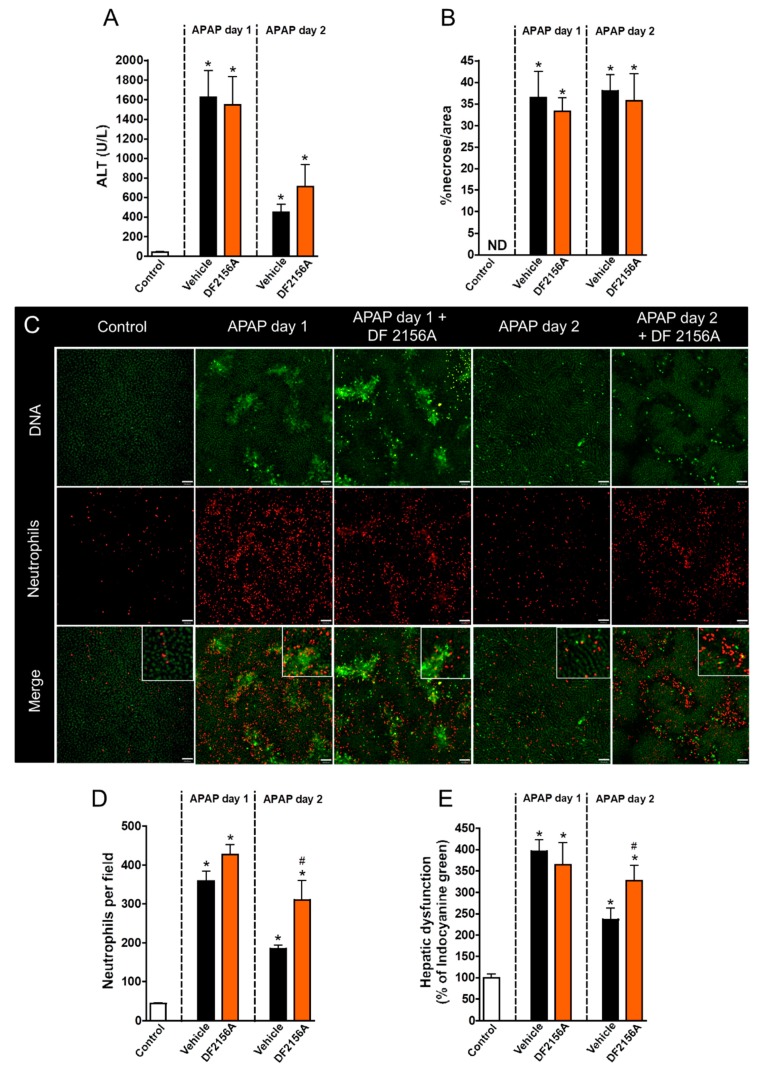
CXCR1/2 antagonism delayed liver repair. (**A**) Serum ALT levels evidencing liver injury. (**B**) Liver necrosis morphometry. (**C**) Intravital confocal microscopy showing liver injury sites and neutrophil accumulation in liver parenchyma. (**D**) Quantification of neutrophils in liver parenchyma. (**E**) Hepatic dysfunction assessed by depuration rate of ICG. Green—Sytox Green (5 nmol/mouse); red—phycoerythrin-conjugated anti-Ly6G (4 µg/mouse). Scale bar = 50 µm. * *p* ≤ 0.05 vs. control; # *p* ≤ 0.05 vs. vehicle (mean ± SEM).

CXCR1/2 chemokine receptors are the most well studied receptors in neutrophil activation and recruitment through recognition of CXCL1/2 in mice [13] and CXCL8 in humans [14,15]. To investigate whether blocking neutrophil migration to the liver mimicked the effects of MMP inhibition, we treated APAP-challenged mice with DF2156A, an allosteric non-competitive antagonist of CXCR1/2 receptors 12 h after APAP administration (30 mg/kg, i.v.). We found that CXCR1/2 antagonism did not prevent liver injury because no changes in serum ALT levels (Figure 7A) and liver necrosis (Figure 7B) were observed after DF2156A treatment. However, CXCR1/2 antagonism caused a massive retaining of neutrophils within the liver microenvironment, particularly within necrotic/injured areas (Figure 7C,D). Interestingly, similar to what we observed during MMP inhibition, blockage of CXCR1/2 also caused a significant delay in hepatic recovery after APAP intoxication (Figure 7E). Thus, while neutrophil infiltration and MMP release within the liver may be associated with amplification of liver injury and inflammation, the recovery phase is dependent on these initial phenomena triggered by APAP-mediated cell death.

## 4. Discussion

Neutrophils are essential to host defense during infections and it is becoming more evident that the collateral tissue damage caused by these cells is a consequence of their primary effector function. In fact, injured tissues must be repaired to ensure proper functioning and it is assumed that the primary purpose of inflammation is to restore tissue homeostasis. However, a clear demonstration of the dual role of neutrophilic inflammation remained elusive in the literature. Here we described the dynamics of neutrophil roles in both liver injury and tissue repair after a sterile liver injury. In this sense, neutrophil depletion or impairment of different neutrophil effector functions 12 h after APAP challenge increased liver injury, which was associated with delayed tissue repair and the restoration of liver function.

Here we expanded on our previous findings that established the dynamics of neutrophil accumulation within the liver parenchyma after APAP challenge [16]. We showed that both neutrophil influx and liver injury peaked 12 h after APAP challenge, suggesting that neutrophils may have a role in amplifying APAP-induced liver injury. The presence of neutrophils in the liver parenchyma after APAP-induced liver damage was first reported in 1973 by Mitchell et. al. [17] and subsequent work showed that neutrophil depletion attenuated liver injury induced by APAP [18]. However, neutrophil depletion by anti-GR1 administration appeared to provide APAP toxicity resistance regardless of neutrophil depletion [19]. Treatment with an RB6-8C5 clone (the most common commercially available anti-GR1 antibody) causes the depletion of neutrophils, monocytes and other leukocytes [20], making data interpretation very challenging. This may be attribute to its effects in both Ly6G and Ly6C antigens. Furthermore, it has been reported that anti-GR1 treatment can dampen APAP toxic effects in a mechanism independent of the immune system [21]. Here we induced neutropenia using a specific anti-Ly6G antibody (clone 1A8) and showed that neutrophil depletion not only worsened liver function, but also delayed liver tissue repair. This corroborates the idea that neutrophils not only trigger all initial steps of acute inflammation [22], but also orchestrate the resolution of the inflammatory process. Consistent with this, there is a growing body of evidence suggesting that neutrophils play a fundamental role in the reparation phase of inflammation by producing pro-resolutive mediators such as lipoxins [23], scavenging pro-inflammatory cytokines [24,25] and the pro-resolutive mediator AnxA1 [26].

Neutrophils are well known for their large stock of intracellular granules. These organelles have different contents and functions but, in a broad sense, neutrophilic granules mainly have tissue-degrading enzymes with a powerful bactericidal effect. In fact, these enzymes have been associated with enhanced tissue damage, pus formation and propagation of tumor cells to remote sites [27]. We showed that unspecific blockage of neutrophil degranulation, or broad-spectrum inhibition of neutrophil granular contents, dampened liver injury after APAP challenge. This suggests that neutrophil degranulation amplifies a sterile inflammatory response. Using a complex pharmacological approach, we showed, amongst different granule enzymes, that matrix metalloproteinases are not only involved in the pathogenesis of liver injury induced by APAP administration, but also crucial for the resolution of the inflammatory process, because inhibition of neutrophil degranulation or MMP activity delayed liver tissue repair. Although MMPs are classically known for their role in tissue disruption by degradation of the extracellular matrix, several MMPs may also have a potent anti-inflammatory function. In fact, different chemokines are metabolized by MMPs within inflamed tissues. For instance, C–C motif chemokines, as well as CXCL12, can be cleaved by MMP2 [28] and CXCL1 can be inactivated by MMP9 [29], demonstrating that while chemokines and other inflammatory mediators are released in response to injury to attract potentially harmful neutrophils, their intracellular contents might also be involved in the resolution of the inflammatory process.

Altogether, our findings highlighted important features of the dynamics of the sterile inflammatory response. Initial massive necrosis triggered by toxic doses of APAP caused oncosis and rupture of the hepatocytes [30,31]. Intracellular damage-associated molecular patterns (DAMPs), including ATP, mitochondria-derived formyl peptides, and DNA, elicit a robust inflammatory response by means of the release of several chemokines within the liver. These mediators rapidly attract neutrophils to sites of necrosis and, probably activated by the same DAMPs, these cells degranulate and release their intracellular enzymatic contents. These enzymes perform potent tissue degrading activities, which ultimately enhance organ injury. Together, these actions may culminate in organ failure. Despite other cells expressions of MMPs, neutrophils were the main leukocytes that infiltrated the liver after APAP challenge. In line with this, neutrophils expressed higher levels of MMPs suggesting that the effects observed in this study could be mainly derived from the inhibition of MMPs from neutrophils. In fact, it has been suggested that an increased MMPs release during APAP intoxication was associated with liver damage and microcirculatory dysfunction. In these cases, an impaired sinusoidal perfusion and infiltration of erythrocytes in Disse space enhanced liver dysfunction during APAP-mediated injury. All these features were minimized when MMPs were inhibited by 2-[(4-Biphenylsulfonyl) amino]-3-phenyl-propionic acid, suggesting that MMPs may play a crucial role in APAP hepatotoxicity [32]. However, once the acute inflammatory response is finished, we propose that necrotic tissues need to be cleared and removed to leave room for cell proliferation and tissue repair. These later phase phenomena can be mediated by the same enzymatic scheme involved in the acute response, since dead or dysfunctional cells need to be digested and phagocytosed prior to total organ healing. In this context, inhibition of neutrophil infiltration or the blockage of key effector molecules including MMPs and other granular enzymes may preclude these cells to exert such “tissue clearing” activity. Retention of debris or delay in removal could be associated with a longer period of full recovery of the organ’s normal architecture and function. It is worth noting that liver recovery as a result of hepatocyte protection by neutrophils and a neutrophils ability to activate liver regeneration are still under debate. We believe that the final phenotype could be a result of a combination of both phenomena, culminating in the lower injury levels observed in our mice.

## 5. Conclusions

Neutrophil infiltration within an inflamed liver is associated with enhanced necrosis which can be partially mediated by MMP activity within the inflamed tissues. However, the initial inflammatory reaction may be crucial to dead cell and debris removal, allowing for full recovery of organ function, as demonstrated by our pharmacological interventions. Neutrophils seem to exert these functions via a release of granular enzymes, mainly MMPs. Our data begets that therapies aimed in controlling the features of acute inflammation need careful evaluation since the effective return to physiological function may be dependent on the full execution of the first steps of the immune response.
